# On Punch, as a Substitute for Wine in Typhus

**Published:** 1800-03

**Authors:** 

**Affiliations:** Fort George


### *To the Editors of the Medical and Phyſical Journal.*

GENTLEMEN,

IF the following trifles are conſiſtent with the plan of your work, they are at your ſervice.

*Fort George, Jan.* 14, 1800. VIATOR.

---

Among thoſe remedies of which modern experience has either diſcovered or extended the uſe, wine ſtands pre-eminently conſpicuous. None is oftener, or more efficaciouſly employed; it is a grateful and ſalutary cordial for every ſpecies of debility, and oppoſes the ravages of fever with unequalled power; but great as its virtues are, we are too often obliged to forego them, from the inability of the patient to provide himſelf with ſo expenſive an article: A thing the more to be regretted, ſince the caſes requiring the uſe of wine are innumerable, and the effects which a phyſician has it in his power to produce by it, altogether aſtoniſhing; as typhus, the bane of all manufacturing and populous towns, is often not at all, and never quickly, to be removed but by this remedy.* Diluted ſpirits have been ſubſtituted, as many ſay, with ſucceſs; and Cullen, if I be not miſtaken, thought at one time, that punch and opium were not inferior to wine. I find, however, that in Edinburgh, (I do not know whether in other medical ſchools alſo) this opinion has fallen, like many of its predeceſſors, into neglect, ſome experiments made in the infirmary there having proved hoſtile to it. A few trials, however, cannot be allowed to decide this point, which the price of wine has made of conſequence; and I am indeed deceived, if I have not often ſeen excellent effects from the adminiſtration of alcohol. Much reaſoning has been uſed to explain the imagined difference of operation between theſe two remedies. The tartareous acid and aſtringent matter have each their advocates, and there has been hitherto a ſort of myſtery about this affair. Tartareous acid will not cure fever, neither will aſtringents, nor the extractive matter, nor alcohol, nor water; not even a mixture of all theſe is ſaid to ſucceed. But wine does; and yet wine is nothing but a mixture of the ingredients juſt enumerated. This

---

* I do not here advert to the yet unconfirmed uſe of the affuſion of cold water, propoſed in a moſt ingenious work by Dr. Currie. If a nameleſs man may ſpeak of his own very limited experience, he would ſuggeſt the inquiry, whether the affuſion, if unſucceſsful, be not very apt to produce obſtinate diarrhœa.

seems intelligible only in one of two ways: Either there is some chemical difference between the alcohol in wine, and the pure, or only contaminated with water and empyreumatic oil; for acids, we know, operate a change on the component parts of alcohol; and thus also, the ardent spirit existing in wine, may differ from that distilled in some proportion of hydrogen, or carbon, or otherwise, but be converted by the heat into common alcohol; or, if this be not the case, the difference must arise from the mode of union of the ingredients. In wine, there is a perfect combination of materials; in diluted alcohol or punch, perhaps little more than an apposition, at least an union in some respect less perfect. Whoever has mixed alcohol and water, must have observed the spirits like threads through the liquor; and this appearance it is difficult to remove entirely, marking rather a diffusion than an union. I know, myself, a gentleman who can readily distinguish two spirits of equal strength, of which the one is pure from distillation, and the other prepared by the admixture of water with a spirit of superior strength. There must be, surely, some distinction, when the taste can discriminate. Many other substances will not unite by simple apposition, without heat or trituration, as sulphuric acid and mercury, and multitudes of others; and it seems doubtful, whether in punch, as commonly made, there be any real union of the parts. But grant this, it may be said, and what follows? Every body knows that while a glass or two of wine strengthens the body, a bottle or two weakens it; and that, in typhus, a large quantity through the day, in small doses, does much good, which quantity taken at once, does a great deal of harm. So wine, being a homogeneous fluid, excites a gradual and gentle action, while punch being a heterogeneous fluid, acts partly as water, and partly as alcohol: that is, one part is inert, and the other acts with the violence of spirits, and so tends to debilitate, as that substance always does. If this be true, by a more perfect combination of alcohol with water, a substance might be produced, at least more approaching to the qualities of wine. Punch is now prepared by a hasty mixture of the ingredients, and is used as soon as made; but to exert its full effect, more care is necessary: hot water should be used, and the liquor afterwards frequently agitated; and the longer it is kept, if secured from evaporation, so much the better. I should suppose, that if a slight fermentation were induced, the mixture would receive material advantage. If this were true, it would be of immense advantage to the poor, who can often afford this, when wine is beyond their reach; and the large hospitals would save part of an enormous expence, now incurred for wine. I am the more inclined to think there is some foundfoundation for this opinion; becaufe, the home-made wines are admitted to be ufeful in medicines, and are certainly only the ingredients of punch more completely combined.

